# International Clinical Practice Guidelines on Traditional Chinese Medicine Therapy for Dysmenorrhea: Development Protocol

**DOI:** 10.2196/77423

**Published:** 2026-03-11

**Authors:** Li Zhang, Xiao Liang, Junlu Chen, Yuhan Du, Huilan Du, Xiaohui Wang

**Affiliations:** 1 First Clinical School (Affiliated Hospital), Shaanxi University of Traditional Chinese Medicine, Xianyang, Shaanxi Province, China; 2 Collaborative Innovation Center of Integrated Chinese and Western Medicine on Reproductive Disease, Hebei Key Laboratory of Integrative Medicine on Liver-kidney Patterns, College of Integrated Traditional Chinese and Western Medicine, Hebei University of Chinese Medicine, No 326, Xinshi South Road, Qiaoxi District, Shijiazhuang, Hebei, 050091, China, 86 13931150880; 3 Graduate School, Hebei University of Chinese Medicine, Shijiazhuang, China; 4 School of Public Health, Lanzhou University, Lanzhou, China

**Keywords:** endometriosis, adenomyosis, dysmenorrhea, traditional Chinese medicine, international clinical practice guidelines, protocol

## Abstract

**Background:**

Traditional Chinese medicine (TCM) functions according to the concepts of “holism” and treatment based on syndrome differentiation, and it has achieved good clinical results in treating patients with dysmenorrhea, which is a common gynecological disorder. However, there are currently no international clinical practice guidelines involving TCM therapies for dysmenorrhea. This study aims to establish a protocol for the development of such guidelines.

**Objective:**

This protocol will provide a road map for the development of the first international clinical practice guidelines on TCM therapy for dysmenorrhea.

**Methods:**

The guidelines will be developed with reference to the *General Rules of Preparation of Diagnosis and Treatment Guideline in Traditional Chinese Medicine* and the *Western Medicine Guideline and Traditional Chinese Medicine Guideline: Improving Together in Mutual Learning*. They will be developed in accordance with the Appraisal of Guidelines for Research and Evaluation II and the World Health Organization guideline handbook and will make recommendations based on systematic reviews. We have established a guideline working group and will formulate clinical questions using the participants, interventions, comparisons, outcomes, and study design framework. The recommendations will be developed through evidence retrieval, synthesis, and the Delphi method. We will consider the preferences and values of patients, as well as the costs and the pros and cons of interventions.

**Results:**

This work was supported by the National Key R&D Program of China (grant 2019YFC1712000) in 2019. The writing group for guidelines was formed in January 2021. The literature search and screening process began in May 2022. So far, the literature search and 2 rounds of the Delphi method have been completed. The protocol will provide a road map for the development of the first international clinical practice guidelines on TCM therapy for dysmenorrhea.

**Conclusions:**

The guideline will be developed in accordance with this protocol, which may provide support and evidence for TCM treatment in patients with dysmenorrhea. There is currently a need for clinical practice guidelines in TCM for the treatment of dysmenorrhea.

## Introduction

### Description of Dysmenorrhea

Dysmenorrhea is a common disorder characterized by painful cramps that occur with menstruation and lower abdominal pain, and it may be accompanied by other symptoms such as nausea, diarrhea, headache, and lightheadedness. The prevalence of dysmenorrhea is highest in adolescent girls, with estimates ranging from 45% to 97% in women of different ages and nationalities [1]. However, only a small percentage of women with this disorder consult a physician about their condition, and many choose to self-medicate [2]. Up to 15% of women with dysmenorrhea have symptoms that are severe enough to cause absences from school or work, as well as restrictions on social, academic, and sports activities [3]. Dysmenorrhea is often underreported and undertreated, and proper dysmenorrhea management will improve the quality of life and alleviate the academic and financial burdens of many women [4].

Dysmenorrhea is classified as primary or secondary dysmenorrhea according to the identification of the underlying cause [5]. Primary dysmenorrhea is defined as pain without any organic cause and is most common in women aged <25 years, with pain beginning within 3 years after menarche. The duration of the pain is commonly 8 to 72 hours and is typically related to the onset of menstrual cramps. In contrast, secondary dysmenorrhea is more likely to occur at any time after menarche but may appear as a new symptom during a woman’s third and fourth decades, after the onset of an underlying causative condition [6]. The pain is related to conditions such as leiomyomas, endometriosis, and pelvic inflammatory disease [3]. The physiological causes of dysmenorrhea include increased levels of prostaglandin in the menstrual fluid [7] and overproduction of vasopressin, a hormone that stimulates uterine muscle contraction [8]. Secondary dysmenorrhea is caused by specific pelvic pathology, and its treatment focuses on the causative pelvic pathology or medical diseases [3]. Risk factors for dysmenorrhea include heavy menstrual flow, nulliparity, depression, and smoking [9].

### Description of Interventions

There are multiple treatment options for dysmenorrhea, many of which have reasonable efficacy and safety profiles. Therefore, there is no one-size-fits-all algorithm for the treatment of dysmenorrhea. Specific factors, including use of contraception, contraindications, ease of use, patient preferences, potential side effects, and cost, can help guide the choice of initial treatment [3]. Nonsteroidal anti-inflammatory drugs and hormonal contraceptives are considered first-line treatments for dysmenorrhea, and they provide effective pain relief for most women [10]. However, the long-term use of nonsteroidal anti-inflammatory drugs and hormonal contraception may have adverse effects, and some patients have contraindications to the use of these medications, including a history of thrombosis or digestive problems. In addition, approximately 10% to 18% of women affected by dysmenorrhea do not respond to these medications. In all these cases, women seek alternatives, including vasodilators, antispasmodics, oxytocin, and vasopressin receptor antagonists, as well as complementary or alternative therapy [11]. The use of complementary or alternative therapy has been welcomed by both consumers and mainstream medical practitioners [6]. These interventions include heat, Chinese herbal medicine, acupuncture, transcutaneous electrical nerve stimulation, yoga, and exercise [12].

Traditional Chinese medicine (TCM) is considered to be a viable option for dysmenorrhea treatment. It has been used in China and many countries in Southeast Asia for a long time. In recent years, it has been used worldwide due to its lack of side effects, safety, and efficacy. TCM uses herbal medicine combined with acupuncture, massage, and acupoint stimulation to treat disease, and these approaches have demonstrated therapeutic benefits. However, there is a worldwide lack of clinical research and clinical practice guidelines for TCM treatment of dysmenorrhea.

### Principles of TCM

Dysmenorrhea is a common gynecological disorder, with qi-blood circulation running not fluently, deficiency of both qi and blood, or asthenia of liver and kidney as the main pathogenetic factors, and the disease locations are the uterus and the thoroughfare-conception meridian. Dysmenorrhea occurs along with the menstrual cycle and is related to the specific physiological state of the menstrual period itself, as well as before and after the menstrual period, that is, during the period of nonmenstruation. The pathogenic factors and the insufficient qi-blood harmonization lead to qi stagnation and blood stasis or insufficiency. Before and after menstruation, blood changes from fullness to overflow, during which time the body becomes more vulnerable to pathogenic influences. This may result in impaired circulation of qi and blood or underlying qi-blood deficiency. The treatment is based on the principle of coordinating the thoroughfare-conception meridian and harmonizing the qi-blood. During the menstruation period, the emphasis is on promoting blood circulation for removing blood stasis and relieving pain to achieve symptom relief. The medicine is usually taken 3 to 5 days before the pain is expected and is continued until the pain is relieved. The emphasis is on treatment upon syndrome differentiation to achieve resolution of the symptoms in chronic cases. This requires continuous treatment for more than 3 menstrual cycles.

### Syndrome Differentiation of Dysmenorrhea in TCM

Treatment upon syndrome differentiation is the TCM principle used for the recognition and treatment of diseases. Syndrome differentiation should be based on the time of occurrence of dysmenorrhea and the nature and degree of the pain, combined with menstrual conditions and systemic syndromes to distinguish patterns of cold vs heat and deficiency vs excess. TCM usually classifies dysmenorrhea into the following syndromes: (1) qi stagnation and blood stasis syndrome, where delayed qi movement causes disorders of blood circulation, with the pathological changes coexisting with qi stagnation and blood stasis; (2) deficiency of both qi and blood syndrome, characterized by pathological changes in parts of the body that are deprived of nourishment and thus have reduced function; (3) cold congealing and blood stasis syndrome, where cold causes congealing, affecting qi and causing the blood to stagnate, and where the meridians are blocked; (4) cold damp stagnation syndrome, where, due to the intrusion of cold and moisture, the moisture cannot be excreted smoothly, resulting in blood stasis symptoms; (5) liver-kidney depletion syndrome, where there is deficiency in the liver and kidney, resulting in deficiency of the thoroughfare-conception meridian, and where the sea of blood is empty, and the uterus and thoroughfare-conception meridian lose nourishment; (6) damp-heat stasis syndrome, where accumulated heat and damp rush into the uterus, the circulation of qi and blood is blocked, the sea of blood before menstruation is full of qi and blood, the heat and damp are intertwined with blood, and stagnation cannot be overcome; and (7) yang deficiency and internal cold syndrome, where there is weakness of the kidney yang, endogenous cold pathogen, loss of warmth in the uterus, and impaired circulation of qi and blood.

### Objective

This protocol aims to provide a road map for the international guideline for the diagnosis and treatment of dysmenorrhea in TCM. There is a worldwide lack of clinical research and clinical practice guidelines for the TCM treatment of dysmenorrhea. These guidelines would represent the first international clinical practice guidelines on TCM therapy for dysmenorrhea and will provide substantial evidence and standardized guidance for treating dysmenorrhea using TCM. As this guideline aims to develop a TCM symptom guideline for dysmenorrhea, it is divided into primary and secondary dysmenorrhea (endometriosis and adenomyosis) for research.

## Methods

### Principles

The study will reference the *General Rules of Preparation of Diagnosis and Treatment Guideline in Traditional Chinese Medicine* [13-15] and the *Western Medicine Guideline and Traditional Chinese Medicine Guideline: Improving Together in Mutual Learning* [16,17]. It will follow the Chinese Clinical Guidelines Evaluation System (AGREE-China) [18-20] and the World Health Organization guideline handbook [21] and will make recommendations based on systematic reviews. We have established a guideline working group and will formulate clinical questions using the participants, interventions, comparisons, outcomes, and study design (PICOS) framework. The recommendations will be developed through evidence retrieval, synthesis, and the Delphi method. We will also consider the preferences and values of the patients, as well as the cost and the pros and cons of the interventions. The guideline has been registered on the International Practice Guidelines Registry Platform (registration number IPGRP-2021CN138).

### Guideline Development Institutions, Target Users, and Population

The guidelines will be developed by Hebei University of Chinese Medicine. The Evidence-Based Medicine Center of Lanzhou University will provide methodological support. The target users of the guidelines are Chinese and Western medicine obstetricians and gynecologists, TCM physicians, physicians of integrated Chinese and Western medicine, nursing staff, and Chinese pharmacists. The target population is patients who are clinically diagnosed with primary dysmenorrhea or endometriosis or adenomyosis.

### Guideline Working Group

The guideline working group was established in June 2021 and consists of 6 subgroups: the guideline steering committee, conflict of interest management committee, consensus expert group, secretary group, evidence evaluation group, and external review group.

The steering committee consists of 7 members, including 2 masters of TCM, 4 gynecologists practicing TCM, and 1 expert in evidence-based medicine. The guideline steering committee will be mainly responsible for (1) determining the topic and scope of the guideline; (2) establishing other working groups for the guideline; (3) managing conflicts of interest; (4) approving the guideline plan, recommendations, and full text; (5) supervising the development process of the guideline; (6) documenting the guideline development process; (7) writing and submitting the first draft of the guideline; and (8) monitoring and evaluating the published guideline.

The conflict of interest management committee is composed of 3 members who will manage, evaluate, and approve the declaration of interest at each stage of the guideline formulation. The main principles are as follows: (1) as far as possible, members with conflicts of interest should not be included; (2) all participants in the guideline development process will be required to declare their own and their family members’ conflicts of interest and continue to update them before participating in any link; and (3) the conflict of interest statement should report all possible conflicts of interest in the previous 3 years.

Taking into account the balance of disciplines, majors, gender, and geographical distribution, we established a consensus expert group, which is composed of 30 experts in related fields, including 2 methodological experts, 16 Chinese medicine experts, 10 Western medicine experts, and 2 pharmaceutical experts. This group is mainly responsible for the determination of clinical issues, conducting consensus and votes on recommendations, and the finalization of the full text of the guidelines.

The secretary group consists of 5 members. These 5 members are mainly responsible for (1) coordinating the work of other working groups, (2) drafting the guideline plan, (3) conducting research into clinical problems, (4) organizing recommendation consensus meetings, (5) recording the entire process of guideline formulation in detail, and (6) writing the first draft of the guideline and submitting the guideline.

The evidence evaluation group consists of 7 members. The 7 members are mainly responsible for (1) retrieving, evaluating, synthesizing, and grading evidence; (2) the production of a systematic review; (3) the production of evidence summary tables; and (4) the production of recommendation and opinion decision tables.

The external review group is composed of 5 members who are mainly responsible for reviewing the final version of the guideline; ensuring its scientific worth, clarity, and fairness; and giving feedback and suggestions on the major risks or problems of the guideline and the content of specific recommendations.

Several foreign guideline development agencies, including the World Health Organization, National Institute for Health and Care Excellence, and Scottish Intercollegiate Guidelines Network [22-24], require the participation of patient representatives. In the guideline formulation process, the participation of patients in the formulation of the scope of the guideline and clinical problems can assist in ensuring that the problems to be solved by the guideline are relevant to the patients [25]. Therefore, according to these requirements of guideline development, the guideline working group recruited 2 patients. The 2 patients participated in raising questions, evaluating evidence, and forming recommendations for the guideline. During the question-raising stage, the guideline development team understood patients’ perspectives on the research topics, helping to ensure that the chosen research questions truly align with clinical practice and patients’ concerns. In the evidence collection and evaluation stage, patients’ opinions were sought to understand their real experiences and needs during actual treatment. In the recommendation formation stage, the guideline development team could directly learn patients’ views and feedback on the recommendations, further clarifying the key factors that should be considered in treatment decisions. In addition, the guideline development team could survey different patient groups to obtain information on variations in treatment preferences, ensuring that the recommendations can meet the needs of different patients. Draft recommendations are published in clear and understandable language, inviting patients to provide feedback to ensure that the recommendations not only align with the latest evidence but also address patients’ real concerns.

### Declaration of Interests

All members of the guideline steering committee, consensus expert group, secretarial group, external review group, and other experts or consultants participating in the guideline development process will be required to fill out the conflict of interest declaration form before participating in the guideline development work to ensure that potential conflicts of interest are declared and disclosed.

### Formulating Questions and Choosing Outcomes

According to the topic of the guideline, the guideline steering committee and secretary group will collect clinical questions from different levels of clinical medical workers in different levels of hospitals and will compile a preliminary list of clinical questions based on the full texts of existing literature and international guidelines. They will categorize clinical issues, deconstruct PICOS issues, conduct 2 to 3 rounds of expert consensus, and will finally determine the clinical issues to be included. The format of the clinical questions will follow the PICOS principles [25,26]. For example: Is Chinese medicine an effective way to treat dysmenorrhea?

The *P* in PICOS stands for population or participants; here, it refers to all patients with primary dysmenorrhea, endometriosis, or adenomyosis. *I* stands for intervention; here, it refers to patients who use Chinese medicine. *C* stands for comparison; here, it refers to patients who do not use Chinese medicine. *O* stands for outcome; here, it refers to degree of pain and adverse reaction.

On the basis of a preliminary analysis of the published literature and clinical practice, the program will select outcome indicators, including the degree of dysmenorrhea; duration of dysmenorrhea; scores from the visual analogue scale, Cox Menstrual Symptom Scale, Dysmenorrhea Symptom Score, Short-Form McGill Pain Questionnaire, pain numerical rating scale, and Cox Retrospective Symptom Scale; serum prostaglandin F2α/prostaglandin E2 levels; uterine hemodynamic indicators; lesion size; menstrual volume; carbohydrate antigen 125 level; patient fertility; recurrence rate; and adverse reactions. These outcome indicators represent the efficacy of dysmenorrhea treatment. Each outcome indicator will be scored on a 9-point scale, with 1 to 3 points indicating that the outcome indicator is not important, 4 to 6 points indicating that the outcome indicator is important, and 7 to 9 points indicating that the outcome indicator plays a vital role in decision-making [25].

### Evidence Retrieval and Synthesis

#### Databases

We will systematically review the literature from MEDLINE, Web of Science, Embase, Cochrane Library, and 4 Chinese databases (CNKI, Weipu, Wanfang, and China Biomedical Literature Database). The date of publication will be restricted from January 1, 2001, to June 30, 2022. The publications will be limited to those published in Chinese and English.

#### Search Terms

The following Medical Subject Headings (MeSH) items and free words will be used to balance the search sensitivity and specificity: dysmenorrhea, primary dysmenorrhea, secondary dysmenorrhea, endometriosis, adenomyosis, herbal, Chinese medicine, traditional Chinese medicine, complementary and alternative medicine, acupuncture, and moxibustion, among others. We will first search for MeSH terms in the MEDLINE database to determine subject terms and free words. On this basis, evidence-based medicine experts from Lanzhou University will provide guidance and develop detailed retrieval strategies. Using PubMed to retrieve “primary dysmenorrhea” as an example, the search process is detailed in Textbox 1.

Textbox 1. **Search terms.**

1. “dysmenorrhea”[MeSH] OR “primary dysmenorrhea”[MeSH]
2. “dysmenorrhea” OR “primary dysmenorrhea” OR “painful menstruations” OR “dysmenorrheas” OR “menstrual pain” OR “painful menstruation” OR “painful period” OR “menstrual pains” OR “menstrual cramp”
3. #3 #1 OR #2
4. “Chinese medicine”[MeSH] OR “traditional Chinese medicine” [MeSH] OR “acupuncture” [MeSH] OR “complementary and alternative medicine” [MeSH] OR “moxibustion” [MeSH] OR “(herbal medicine” [MeSH] OR “TCM” [MeSH]
5. “Chinese medicine” OR “traditional Chinese medicine” OR “acupuncture” OR “complementary and alternative medicine” OR “moxibustion” OR “herbal medicine” OR “TCM” OR “powders” OR “Granules” OR “Decoction” OR “san” OR “Decoction”
6. #4 OR #5
7. “randomized controlled trial” [Publication Type] OR “Meta-Analysis” [Publication Type] OR “Systematic review” [Publication Type] OR “randomized controlled trial” [MeSH] OR “Meta-Analysis” [MeSH] OR “Systematic review”[MeSH]
8. “random” OR “randomly” OR “allocation” OR “blind” OR “concealment” OR “placebo” OR “clinical” OR “RCT” OR “Systematic review” OR “Meta” OR “control” OR “study” OR “trial”
9. #7 OR #8
10. #3 AND #6 AND #9

#### Literature Selection

Randomized controlled trials, systematic reviews, and other types of clinical studies related to this study will be retrieved. After eliminating duplicate and irrelevant studies, the guide secretary group will screen the included literature based on the title and abstract and will then rescreen it by reading the full text to finally determine the selected literature.

#### Evidence Syntheses

High-quality systematic reviews published within the previous 2 years will be used directly. In the absence of relevant systematic reviews, if the quality of existing systematic reviews is not high, or the results of the systematic reviews are of low applicability to the issues addressed by the guidelines, we will consider conducting or updating a systematic review [27].

#### Evidence Assessment

The Grade of Recommendations Assessment Development and Evaluation (GRADE) [28,29] instrument is used for the classification of evidence, including evidence quality classification and recommendation classification. The quality of evidence is divided into very low, low, medium, and high, and the strength of recommendation is divided into 2 levels, weak and strong. Randomized controlled trials are considered high-quality evidence, while observational studies are considered low-quality evidence. Methodologists and clinical experts will jointly adjust the level of evidence based on the evaluation criteria of 5 downgrading factors and 3 upgrading factors to form the final version.

We will evaluate the evidence for the entire study on the basis of different results. The methodologists will be responsible for evaluating the quality of evidence, drafting evidence summaries, and presenting these summaries at the guideline development group meeting. The grading instrument will be used to assess the level of evidence for making recommendations. The evidence evaluation group will develop an evidence summary table, and the guideline development group will divide the relevant evidence for each recommendation into 4 levels based on the scoring instrument: high, medium, low, and very low.

#### Patients’ Preferences and Values

The secretary group will be responsible for investigating the preferences and values of patients with dysmenorrhea in using TCM treatment. The opinions of patients will be fed back to the steering committee and consensus expert group for consideration when formulating recommendations.

#### Developing Recommendations

After completing the evidence classification according to the GRADE system, the preliminary recommendations will be drafted with reference to the paper titled “Criterion and Detailed Judgments for the Transformation From Evidence to Recommendations in the Clinical Practice Guidelines of Chinese Medicine” by Yang et al [30]. Expert consensus will be reached through 2 to 4 rounds of the Delphi method to formulate draft recommendations, which will then be submitted to the steering committee for final approval [31,32]. The GRADE grid structured approach will be used to reach consensus [33]. Each item in the questionnaire will have 5 levels of choice: strongly or weakly recommending for it, making no clear recommendation, and strongly or weakly recommending against it. For each item, if more than 50% of the experts vote for any option other than “no clear recommendation,” or if more than 70% of the experts vote for 1 of the 2 options on the same side, consensus will be deemed to have been reached. If this does not occur, the item will be deemed to have not reached a consensus, and the recommendations will enter the next round of the Delphi process.

#### Drafting the Guidelines

The guidelines will be drafted based on the level of evidence and strength of each recommendation. The reporting specifications of this guideline will refer to the Reporting Items for Practice Guidelines in Healthcare (RIGHT) for TCM [34] during its development. The main text of the guideline will be written at the same time as the corresponding preparation instructions.

#### Peer Review of Guidelines

The draft guidelines will be submitted to the external review group for review and confirmation. The guideline working group will record the review process and collect opinions from the review experts. After fully discussing these opinions, the draft guidelines will be revised appropriately.

#### Publication and Promotion of Guidelines

The guidelines are expected to be published within 1 or 2 years and will be translated into Chinese and English and published in relevant journals. After the guideline is released, it will be promoted in the following ways: (1) the guidelines will be printed as a manual and disseminated in the academic community; (2) the guidelines will be used in lectures at academic conferences; and (3) the Chinese, English, or other language versions of the guidelines will be promoted on popular medical or official health websites or apps.

#### Implementation, Evaluation, and Updating of the Guidelines

The guideline working group will regularly monitor the implementation of the guidelines. This will include process evaluation, implementation effect, performance evaluation, and evaluation of the guideline itself. When there is new relevant evidence or recommendations, or if the strength of recommendations changes after the release of the guideline, the guideline will be updated in a timely manner. The update cycle of the general guidelines will be between 2 and 5 years.

### Ethical Considerations

This study protocol pertains solely to the development of a clinical practice guideline based on a literature review, expert consensus, and analysis of existing, fully anonymized, publicly available, or aggregated statistical data. There is no direct involvement of human subjects, no primary data collection, and no experimental intervention. Expert consultation within this process is for professional opinion formation and does not constitute human subjects research as defined by our institutional policies. Given the nature of the study design, the type of data used, and the minimal risk involved, the study was reviewed and classified as exempt from full ethics review by the Hebei University of Chinese Medicine Science and Technology Ethics Committee.

## Results

This work was supported by the National Key R&D Program of China (grant 2019YFC1712000) in 2019. The writing group for guidelines was formed in January 2021. The literature search and screening process began in May 2022. An initial search conducted in May 2022 across 8 databases (including 4 Chinese databases and 4 English databases) identified 54,029 studies that met the search criteria. Of the 54,029 articles, 51,066 (94.5%) duplicates were removed, leaving 2963 (5.5%) articles for screening. Before screening, an additional search will be conducted to update the identified articles and ensure they are the most current. Two independent reviewers finished the literature search and screening process. After completing the evidence classification according to the GRADE system, the preliminary recommendations were drafted with reference to the paper by Yang et al [30]. Expert consensus was reached through 2 to 4 rounds of the Delphi method to formulate draft recommendations. After 2 rounds of Delphi method expert questionnaire surveys, a consensus among experts was reached, and 41 and 46 feedback responses were collected in the 2 rounds.

## Discussion

### Anticipated Findings

The establishment of the first international clinical practice guidelines on TCM therapy for dysmenorrhea will provide a basis for such therapy. The program aims to adopt an appropriate approach to the development of the guideline. The guidelines will be developed in accordance with this protocol, which may provide support and evidence for TCM treatment for patients with dysmenorrhea. Chinese herbal medicine, acupuncture, massage, acupoint stimulation, and other TCM methods used solely or in combination for the treatment of dysmenorrhea have demonstrated considerable therapeutic benefits. TCM gives good clinical results for the treatment of dysmenorrhea based on the basic principles of holism and syndrome differentiation.

Dysmenorrhea is a common gynecological condition that is further divided into 2 categories, namely, primary and secondary dysmenorrhea. The guideline will clarify the definition, diagnosis, and TCM syndrome classification for dysmenorrhea and recommend different TCM treatments based on the level of evidence, including Chinese herbal medicine, Chinese patent medicine, acupuncture, and other auxiliary therapies. This guideline will provide substantive evidence and standardized guidance for the treatment of dysmenorrhea using TCM, improve the quality and safety of medical services, and standardize diagnosis and treatment plans for dysmenorrhea.

### Strengths and Limitations

This guideline was developed with the involvement and guidance of methodology experts to ensure methodological rigor and transparency. Data screening and extraction were carried out by 2 independent reviewers, with a third reviewer resolving any discrepancies, thereby minimizing selection and extraction bias. However, some limitations can be anticipated. The exclusion of studies in languages other than Chinese and English may reduce the comprehensiveness of the evidence base due to translation limitations. In addition, including nonrandomized studies may introduce bias. Despite these limitations, this guideline will provide substantial evidence and standardized guidance for the treatment of dysmenorrhea with TCM, improve the quality and safety of medical services, and standardize the diagnosis and treatment of dysmenorrhea.

### Future Directions

In the future, we will continue to pay attention to research progress on the treatment of dysmenorrhea with TCM, continuously update the guidelines, and provide guidance for clinical TCM treatment of dysmenorrhea.

### Conclusions

This plan proposes the specific steps for developing guidelines, aiming to establish international clinical practice guidelines for the treatment of dysmenorrhea with TCM and providing guidance for clinical practice. The findings will be disseminated through professional clinical and academic networks, ensuring that research findings are translated into practice and guiding future research directions.

## Abbreviations

GRADE: Grade of Recommendations Assessment Development and Evaluation
MeSH: Medical Subject Headings
PICOS: participants, interventions, comparisons, outcomes, and study design
RIGHT: Reporting Items for Practice Guidelines in Healthcare
TCM: traditional Chinese medicine
